# Comparative modeling of DNA and RNA polymerases from *Moniliophthora perniciosa *mitochondrial plasmid

**DOI:** 10.1186/1742-4682-6-22

**Published:** 2009-09-10

**Authors:** Bruno S Andrade, Alex G Taranto, Aristóteles Góes-Neto, Angelo A Duarte

**Affiliations:** 1 Departamento de Ciências Biológicas, Universidade Estadual de Feira de Santana, Feira de Santana, Brazil; 2 Departamento de Saúde, Universidade Estadual de Feira de Santana, Feira de Santana, Brazil; 3 Departamento de Tecnologia, Universidade Estadual de Feira de Santana, Feira de Santana, Brazil

## Abstract

**Background:**

The filamentous fungus *Moniliophthora perniciosa *(Stahel) Aime & Phillips-Mora is a hemibiotrophic Basidiomycota that causes witches' broom disease of cocoa (*Theobroma cacao *L.). This disease has resulted in a severe decrease in Brazilian cocoa production, which changed the position of Brazil in the market from the second largest cocoa exporter to a cocoa importer. Fungal mitochondrial plasmids are usually invertrons encoding DNA and RNA polymerases. Plasmid insertions into host mitochondrial genomes are probably associated with modifications in host generation time, which can be involved in fungal aging. This association suggests activity of polymerases, and these can be used as new targets for drugs against mitochondrial activity of fungi, more specifically against witches' broom disease. Sequencing and modeling: DNA and RNA polymerases of *M. perniciosa *mitochondrial plasmid were completely sequenced and their models were carried out by Comparative Homology approach. The sequences of DNA and RNA polymerase showed 25% of identity to 1XHX and 1ARO (pdb code) using BLASTp, which were used as templates. The models were constructed using Swiss PDB-Viewer and refined with a set of Molecular Mechanics (MM) and Molecular Dynamics (MD) in water carried out with AMBER 8.0, both working under the ff99 force fields, respectively. Ramachandran plots were generated by Procheck 3.0 and exhibited models with 97% and 98% for DNA and RNA polymerases, respectively. MD simulations in water showed models with thermodynamic stability after 2000 ps and 300 K of simulation.

**Conclusion:**

This work contributes to the development of new alternatives for controlling the fungal agent of witches' broom disease.

## Background

The filamentous fungus *Moniliophthora perniciosa *(Stahel) Aime & Phillips-Mora is a hemibiotrophic Basidiomycota (Agaricales, Tricholomataceae) that causes witches' broom disease of cocoa (*Theobroma cacao *L.). It has been claimed as one of the most important phytopathological problems that has afflicted the Southern Hemisphere in recent decades. In Brazil, this phytopathogen is endemic in the Amazon region [1]. However, since 1989, this fungus has been found in the cultivated regions in the state of Bahia, the largest production area in the country. The fungus caused a severe decrease in the Brazilian cocoa production reducing Brazil from the second largest cocoa exporter to a cocoa importer in just few years [2].

Plasmids are extragenomic DNA or RNA molecules that can independently reproduce in live cells. Their structure can be circular or linear, and include complete protein coding genes, pseudogenes, non-protein coding genes and inverted repetitive elements. The probable plasmid function in their fungal hosts is related to the change of aging time. Fungal linear mitochondrial plasmids present the same basic structure as in other organisms, but they also carry viral-like DNA and RNA polymerase (DPO and RPO, respectively) ORFs and have 3' and 5' inverted terminal repeats, also a 5' binding protein. This protein can be involved in both replication and integration processes of these plasmids in the mitochondrial genomes [3,4]. Interestingly, a linear mitochondrial plasmid with the same typical characteristics carried by the other mitochondrial plasmids was found to be completely integrated in the *M. perniciosa *mitochondrial genome, by the Witches' Broom Genome Project [5].

The Φ29 DNA polymerase is in the group α-DNA-polymerases due to its sensitivity to aphidicolin and specific inhibitors, nucleotides similar to BuAaATP and BuPdGTP [6]. This polymerase is the main replication enzyme of double-strand-DNA viruses from bacteria and eucaryotes. It is a 66 KDa enzyme included in the eucaryotic replicase family [7], able to use a protein as primer in the replication process [8,9]. The T7 RNA polymerase is a 99 KDa single chain viral enzyme that executes a specific-promoter transcription process in vivo and in vitro and is in the single-chain RNA polymerase family. The transcription mechanism carried out by this enzyme shares several similarities with other multichain RNA polymerases [9].

It is generally accepted that the water molecules in the hydration environment around a protein play an important role in its biological activity [10], and contribute to stabilizing the native state of the protein [11]. In addition, this interaction has long been recognized as a major determinant of chain folding, conformational stability, and internal dynamics of many proteins, and as important to the interactions related to substrate binding, enzyme catalysis, and supramolecular recognition and assembly [12]. Standard Molecular Dynamics approaches measure the conformational space of a protein using atomic interactions from several force fields and include explicitly treated water to reproduce solvent effects [13].

The aim of this work to carry out homology modeling of both DNA and RNA polymerases from the linear mitochondrial plasmid of *M. perniciosa*. With the accomplishment of this work, these models can be used as new molecular targets to find drugs against witches' broom disease by de novo design methods [10].

## Methods

After the release of the primary sequences of DNA and RNA polymerases from *M. perniciosa *mitochondrial plasmid, they are available in the Witches' broom project database (LGE). 3D models were built by Comparative Modeling approach. Initially, both DNA and RNA polymerase sequences were subjected to the BLASTp algorithm [14] restricted to the Protein Data Bank (PDB). The templates found were aligned with the protein sequences of both DNA and RNA polymerases by TCOFFEE [15] to find conserved regions and motifs. The 3D models were constructed using SwissPdb Viewer 3.7 [16] following a standard protocol: (I) load template pdb file; (II) align primary target sequence with template; (III) submit modeling request to Swiss Model Server. Then, the initial models constructed by SwissPdb Viewer were prepared using LEAP and submitted to SANDER for structure refinement. The model structures were fully minimized with 100 steps of steepest descent followed by 100 more steps of conjugate gradient to an RMS gradient of 0.01 kcal/2.71Å in vacuum, and then in water for 200 steps of steepest descent followed by 200 more steps of conjugate gradient to an RMS gradient of 0.01 kcal/2.71Å. Next, MD simulations of the refined structures were performed in water using f99 force field at 300 K for 2000 ps. All MD simulations were carried out without constrain methods. The cutoff value of 14 Å was used for minimization of geometry and MD simulations. LEAP and SANDER are utilities of AMBER 9.0 [17,18]. Additionally, all calculations were performed without restraints. Time averaged structures were generated by time averaging of simulations from the point of a stable trajectory, which was obtained through the end of simulation. The Visual Molecular Dynamics (VMD) software [19] was used to visualize trajectory results produced by the SANDER module. Finally, PROCHECK 3.4 [20] and Atomic Non-Local Environment Assessment (ANOLEA) [21,22] were used to evaluate both DNA and RNA polymerases using a Ramachandran plot [23] and energy calculations on a protein chain of each heavy atom in the molecule, respectively [24]. Graphics of RMS × Time were generated by VMD 1.8.6 [25]

## Results and Discussion

Blastp results for both DNA and RNA polymerases of the *M. perniciosa *linear mitochondrial plasmid showed just one reliable template to each enzyme (Table 1). 1XHX [26] and 1ARO [27] were used as template DPO and RPO respectively. Although both of them showed low identity with the targets, it is possible to build useful models for docking studies [10]. The root-mean-squared deviations (RMSD) for Cα between DPO-1XHX and RPO-1ARO are 2.40 Å and 1.84 Å respectively. These values show some differences between models and crystal structures, as one might expect, principally in relation to the number of residues. The models have 543 and 766 residues in DPO and RPO, while the crystal structures have 575 and 883 residues for 1XHX and 1ARO, respectively.

**Table 1:** Selected templates obtained by Blastp algorithm

	**Template**	**Identity**	**E-value**	**Organism**	**RMS (Å)**
DPO	1XHX	32%	8e-06	Phage Φ29	2,40

RPO	1ARO	25%	1e-33	Phage T7	1.84

In addition, these results address the hypothesis of several authors correlating plasmid sequences to DNA and RNA polymerases of adenovirus and retrovirus sequences [3,27].

**Figure 1:**
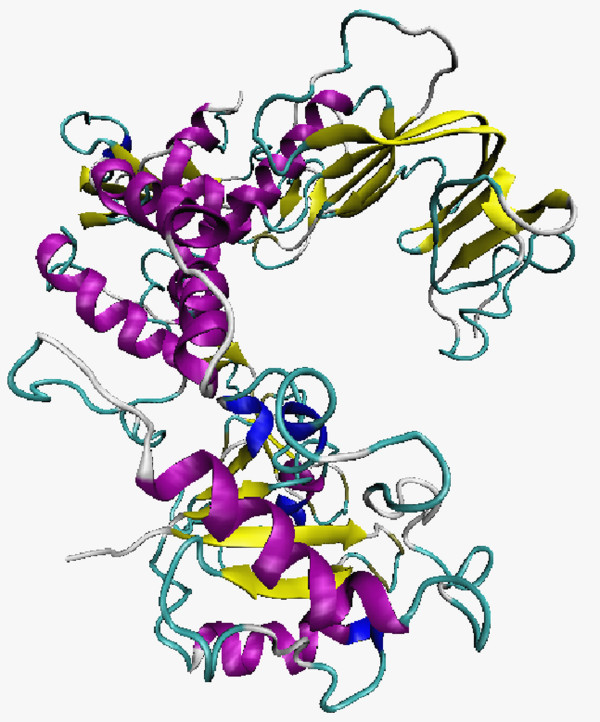
**The 3D structure of the DNA polymerase from the *M. perniciosa *mitochondrial plasmid**. Magenta: helices; yellow: strands; blue: turns.

Using 1HXH as a template, the 3D structure of the DNA polymerase was built from the linear mitochondrial plasmid of *M. perniciosa*. This polymerase was classified within the B family of DNA polymerases, which can be found in viruses and cellular organelles. Figure 1 shows that the DPO model has transferase features with alpha-beta secondary structure.

This model shows 17 alpha-helices, 36 beta-strands, 57 turns, and 315 hydrogen bonds can be observed in the whole structure. As well as other polymerases from that family, this polymerase showed the three standard domains of the group: Palm, Fingers, and Thumb.

**Figure 2:**
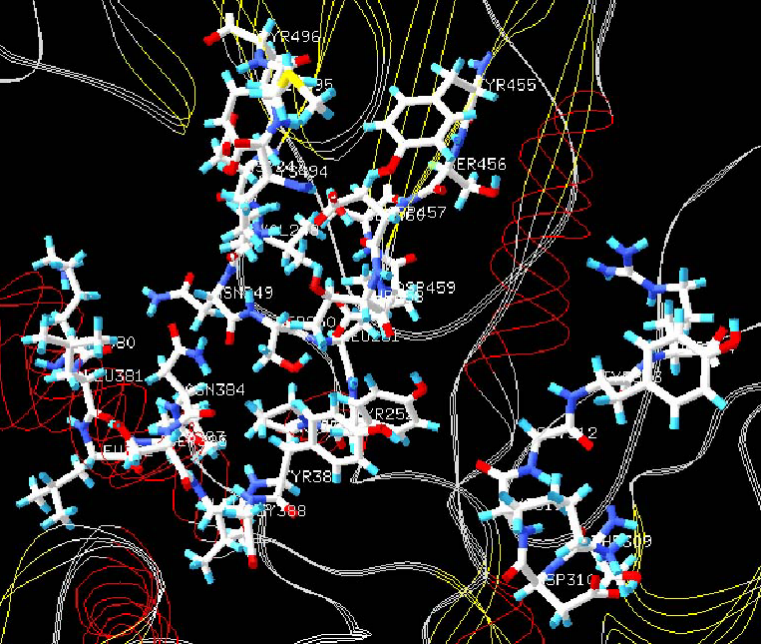
**Active site of the DNA polymerase from the *M. perniciosa *mitochondrial plasmid presenting the conserved motif B**.

The active site of the DNA polymerase of *M. perniciosa *(Figure 2) carries the conserved motif B represented by Lys380, Leu381, Leu382, Leu383, Asn384, Ser385, Leu386, Tyr387, Gly388, and it is involved in dNTP selection and template DNA binding activity as described by Truniger et al. [6] in the homologous Φ29 DNA polymerase. These amino acids are distributed among three domains: Palm, Fingers and Thumb. Other motifs involved with DNA polymerization were found in this polymerase, such as Dx2SLYP (Asp247, Val248, Asn249, Ser250, Leu251, Tyr252, Pro253), YxDTDS (Tyr455, Ser456, Asp457, Thr458, Asp459), Tx2A/GR (Thr309, Asp310, Lys311, Gly312, Tyr313, Arg314) and KxY (Lys494, Met495, Tyr496), which have been reported in several studies [6,8,9,28-31].

**Figure 3:**
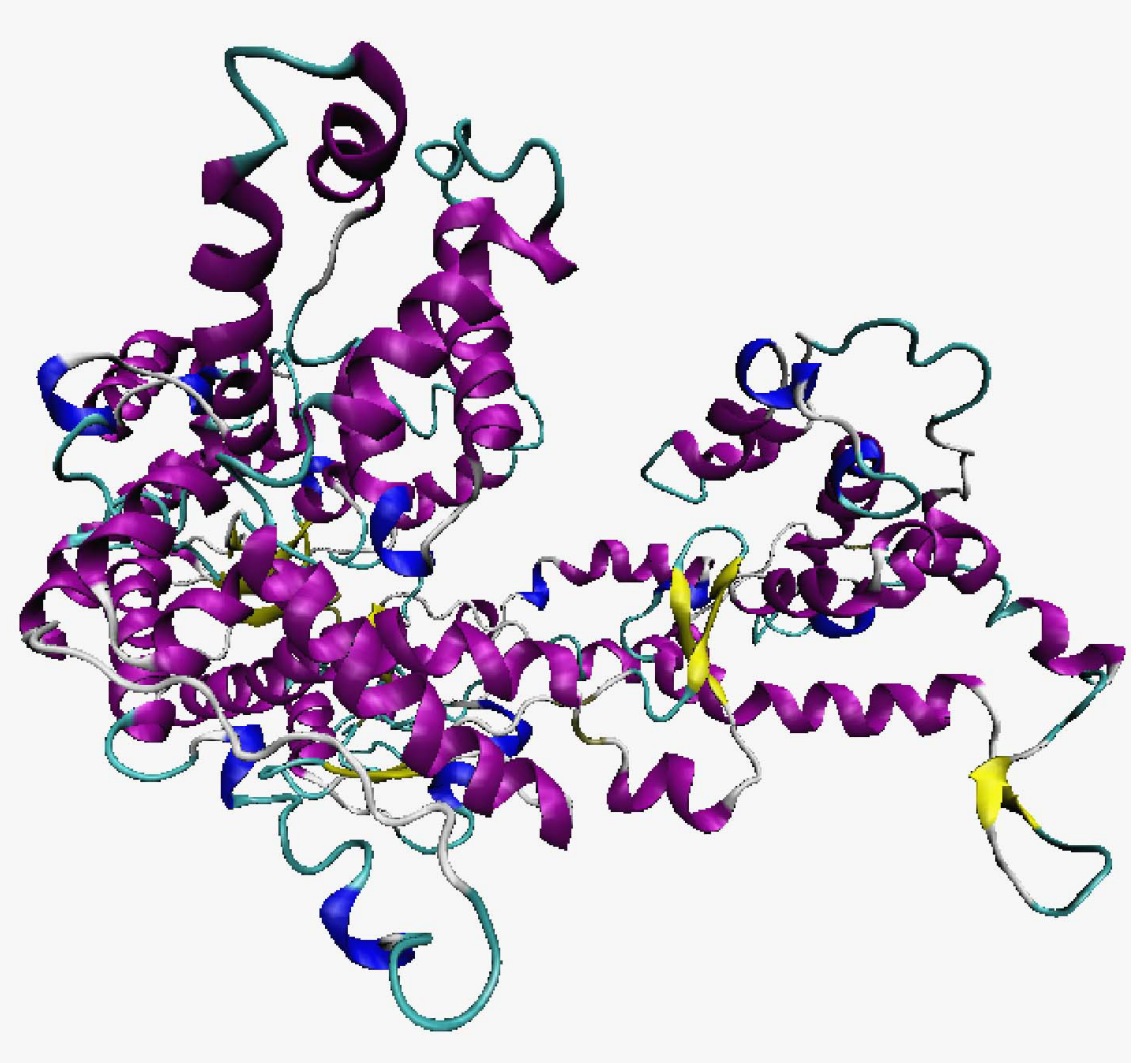
**The 3D structure of the RNA polymerase from the *M. perniciosa *mitochondrial plasmid**. Magenta: helices; yellow: strands; blue: turn.

**Figure 4:**
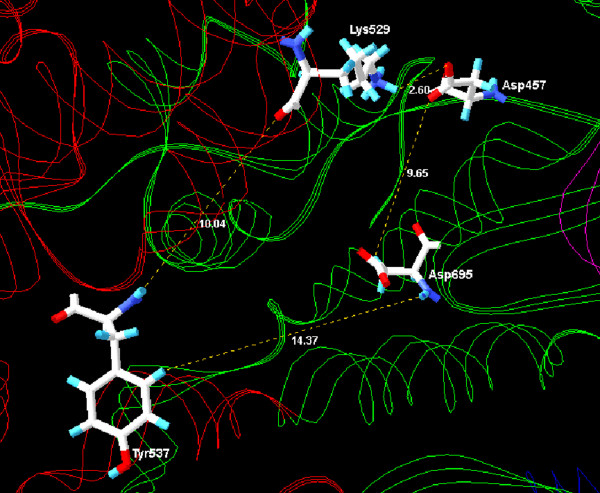
**Active site of the RNA polymerase from *M. perniciosa *mitochondrial plasmid formed by two domains: Palm (green) and Fingers (red)**.

The active site of the RNA polymerase (Figure 3) from *M. perniciosa *plasmid is formed by amino acids from two domains: Palm (Asp457 and Asp695) and Fingers (Tyr537 and Lys529) (Figure 4). In comparison to the template structure, these amino acids perform an alignment in the region of the active site, with the amino acids Asp537 and Asp812 (Palm), and Tyr639 and Lys631 (Fingers) of the template. The presence of these residues (Asp, Tyr, and Lys) in this region is a sign in this group of polymerases that they are involved with transcriptional processes [10,32,33].

**Figure 5:**
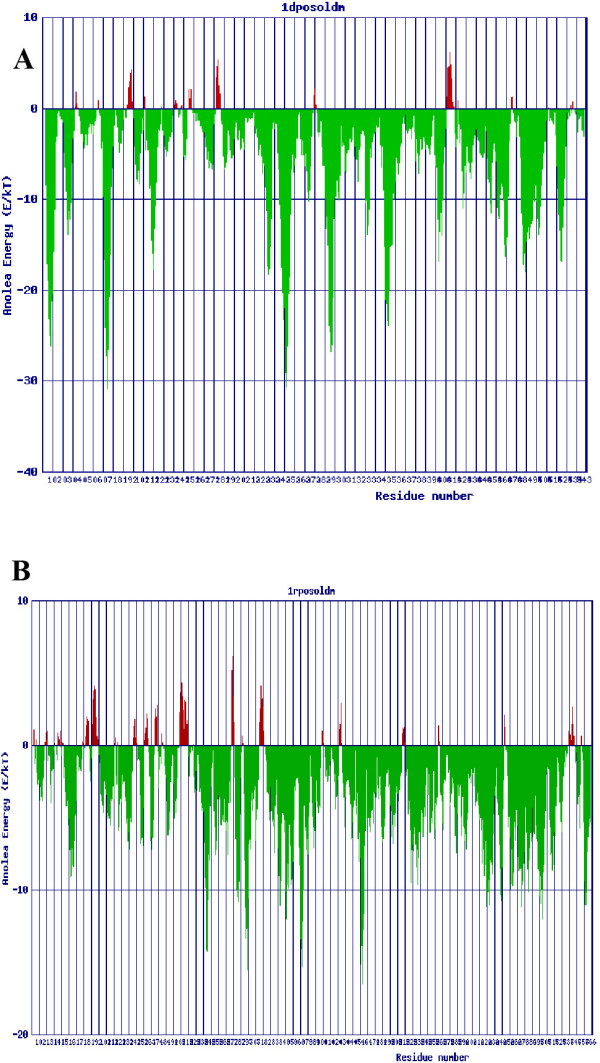
**ANOLEA validation of the built model**. A) DPO; B) RPO. Green and red mean negative and positive values of energy.

Both the DNA and RNA polymerases, after refinement by optimization of geometry and MD simulations, had their structures validated by PROCHECK and ANOLEA (Figure 5). The Ramachandran plot showed that 97% and 98% of residues are within the allowed regions for DPO and RPO, respectively. Almost all residues show negative values of energy (green), whereas few amino acids obtained positive values of energy (red). This means that most residues are in a favourable energy environment. In other words, the quality of both main chain and side chain was evaluated showing that the models had appropriate stereochemical and thermodynamic values. As a result, although the target and template proteins showed a low homology identity, the tertiary structure obtained had the same sign of family.

## Conclusion

The great challenge of genome projects is to elucidate new molecular targets, mainly proteins and enzymes. Functional characterization of proteins is one of the most frequent problems in biology. While sequences provide valuable information, the identification of relevant residues inside them is frequently impossible because of their high plasticity, suggesting a need to construct 3D models. In the case of enzymes, a similar function can be assumed between two proteins if their sequence identity is above 40%. In addition, polymerases are suitable targets for antiviral drugs [34], which have nucleoside analogs as substrates. These inhibitors can be developed by rational design. Thus, our findings address the use of fungi polymerases as starting points for drug design against witches' broom disease, following methodologies similar to those used for the development of inhibitors of polymerases of virus. Our models are suitable for computer aided-drug design approaches, such as docking, virtual screening, and QM/MM in order to search a new lead compound against witches' broom disease.

## Competing interests

The authors declare that they have no competing interests.

## Authors' contributions

BA carried out the templates searching, alignment of target sequences with templates sequences, built the initial models, performed molecular dynamics of the initial models and drafted the manuscript. AT participated in the construction of the initial models, participated in the implementation of molecular dynamics and participated in its design and coordination. AGN participated in the alignment of the sequences of templates with the targets and participated in its design and coordination. AD participated in the implementation of molecular dynamics and participated in its design and coordination. All authors read and approved the final manuscript.
